# Utility, benefits, and risks of newborn genetic screening carrier reports for families

**DOI:** 10.7189/jogh.14.04044

**Published:** 2024-02-23

**Authors:** Xin Wang, Yun Sun, Jing-Yu Zhao, Xian-Wei Guan, Yan-Yun Wang, Dong-Yang Hong, Zhi-Lei Zhang, Ya-Hong Li, Pei-Ying Yang, Tao Jiang, Zheng-Feng Xu

**Affiliations:** 1 Genetic Medicine Center, Women’s Hospital of Nanjing Medical University, Nanjing Women and Children’s Healthcare Hospital, Nanjing, China; 2 Clin Lab, BGI Genomics, Nanjing, China

## Abstract

**Background:**

Newborn genetic screening (NBGS) based on next-generation sequencing offers enhanced disease detection and better detection rates than traditional newborn screening. However, challenges remain, especially around reporting the NBGS carrier results. Therefore, we aimed to investigate the NBGS carrier parents’ views on NBGS and NBGS reports in China.

**Methods:**

We distributed a survey querying demographic information, knowledge and perceptions of NBGS, the impact of NBGS on a total of 2930 parents, and their decision-making to parents of newborns reported as carriers in NBGS in Nanjing, China in 2022.

**Results:**

The average age of the survey respondents was 30.7 years (standard deviation = 3.6). Most (68.38%) felt informed about NBGS, especially women, the highly educated, and high earners. Nearly all (98.74%) saw NBGS as crucial for early disease detection, with 73.18% believing it positively impacts their future. However, 19.16% felt it might cause anxiety, especially among the less educated. Concerns included potential discrimination due to exposed genetic data and strained family ties. Many suggested NBGS coverage by medical insurance to ease financial burdens.

**Conclusions:**

Through our study, we gained insights into parents’ perspectives and concerns regarding the NBGS carrier result reporting, thus providing relevant information for further refinement and clinical promotion of the NBGS project.

Newborn screening is a crucial public health strategy aimed at identifying newborns at early risk of severe genetic disorders [1]. Early identification and intervention significantly enhance health outcomes, help with managing long-term complications, and improve the overall quality of life for affected newborns and their families [2]. Newborn genetic screening (NBGS), based on genomics, represents an advancement over traditional newborn screening [3–6], and by integrating with it, substantially expands the scope of screened diseases, reduces false positive rates, and increases detection rates [5,7–13].

However, ethical debates persist surrounding genetic screening reports due to their involvement with genomic information [14–17], particularly regarding the disclosure of carrier status for pathogenic genes [18,19]. While physicians are obliged to inform families about detected pathogenic information, as understanding carrier status could affect the family’s future reproductive choices and family planning even if newborns do not show signs of the disease [20], disclosure of carrier pathogenic information may lead to unnecessary issues such as privacy breaches and family concerns [21,22].

As parents can directly decide whether to participate in the NBGS project, their perspectives on this issue are crucial for the project's design and implementation. However, existing studies mainly focus on the views and recommendations of clinical professionals and experts within Western populations [10,15,23–26], with limited reporting on the perspectives of carrier parents. Therefore, we aimed to better understand their views on NBGS through a questionnaire survey, but also to explore the necessity of reporting carrier status for NBGS, as well as the benefits, risks, and utility of NBGS for families with newborn carriers, which serves as a robust reference for the smooth implementation of NBGS programmes.

## METHODS

### Study population and sample size calculation

The target population of our survey were couples who gave birth at Nanjing Women and Children’s Healthcare Hospital. Their newborns underwent NBGS testing, with the results indicating carrier status. We calculated the sample size using the Raosoft [27] sample size calculator with a 3% margin of error, 99% confidence level, 50% response distribution, and 8000 as the population size (estimating yearly count of carrier reports based on data from the initial two months of the NBGS programme in our hospital). The minimum required sample size was 1499. During the survey period (18 June 2022 to 18 December 2022), 3981 newborn carrier reports were made accessible to parents through the report inquiry system; we acquired 2390 valid questionnaires for analysis.

### Questionnaire design and validation

We developed a questionnaire centred on the NBGS with 26 specific items (Table S1 in the **Online Supplementary Document**), which we based on insights from prior international newborn genetic screening projects [18,24,26] and professional guidance from clinical genetic counsellors. The questionnaire included four parts: Participants’ basic information such as census register, age, gender, education, and family income; pre-pregnancy and pregnancy check items, as well as reproductive history; questions on participants’ understanding of the genetic diseases, NBGS, and NBGS results; their perspectives and responses to NBGS results, as well as the impact of NBGS results on them.

We have integrated the link to the questionnaire survey into the report inquiry system of the Nanjing Women and Children’s Healthcare Hospital app. To access the NBGS report, family members had to input the barcode number provided during blood collection and the mother's name of the newborn. After reviewing the screening report, participants had to click ‘Next,’ which led them automatically to the questionnaire introduction page containing a brief overview of NBGS and three questions: ‘Are you the parents of the baby?,’ ‘Are you settled in Nanjing, Jiangsu?,’ and ‘Are you willing to participate in this questionnaire survey?’ They could continue with completing the questionnaire only after answering to all three questions.

We initially validated the questionnaire through a pilot analysis for the first 100 responses. We calculated the overall Cronbach’s alpha factor (Q12–23) to ensure the reliability of the questionnaire; the results showed it to be acceptable (raw alpha = 0.62; standard alpha = 0.73).

The Research Ethics Committee of the Women’s Hospital of Nanjing Medical University (2021KY-071) approved this study.

We conducted the statistical analyses in *R*, version 4.1.3. (R Core Team, Vienna, Austria). A *P*-value less than 0.05 was considered statistically significant. We presented categorical variables (Q1–12 and Q17–23) as numbers and percentages, and continuous variables (total scores for Q13–16) as means (x̄) and standard deviations (SDs). We used the χ^2^ test or Fisher exact test to compare groups in terms of categorical variables. As the Shapiro-Wilk test (total scores for Q13–16) showed that the continuous data were non-normally distributed, we used the Mann-Whitney *U* and Kruskal-Wallis non-parametric tests (with Dunn post-hoc test for the latter) to compare two or three and more groups, respectively.

## RESULTS

### Demographic characteristics of respondents

**Figure 1:**
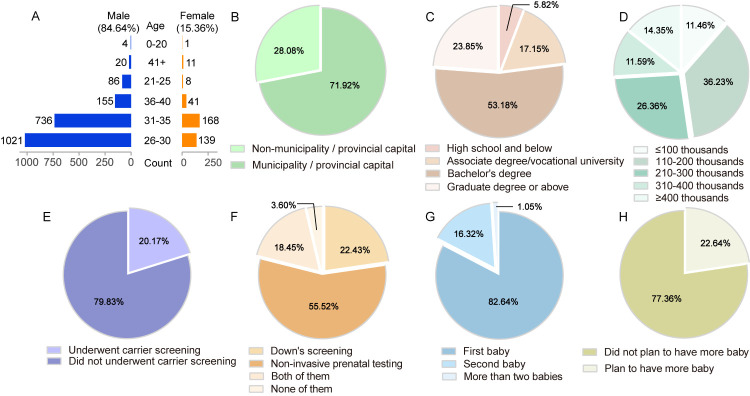
Demographic characteristics of respondents. **Panel A.** Age distribution of respondents. **Panel B.** Geographical distribution of respondents. **Panel C.** Educational level of respondents. **Panel D.** Annual family income of respondents. **Panel E.** Whether they underwent carrier screening. **Panel F.** Whether they underwent Down syndrome screening. **Panel G.** Which baby this was for the respondents (first, second, etc.). **Panel H.** Reproductive plan for respondents.

We received 2390 respondents (84.64% males, age: x̄ = 30.7, SD = 3.6) from parents whose baby was born in Nanjing Women and Children’s Healthcare Hospital. Among the respondents, 71.92% were from the municipality or the provincial capital. Over half (70.33%) had a college degree, while almost half (47.70%) earned <200 000 CNY (**Figure 1** and Table S2 in the **Online Supplementary Document**). Only 20.17% had undergone carrier screening before pregnancy, while just 3.6% skipped Down syndrome or non-invasive prenatal tests (NIPT) during their pregnancy. In terms of family structure, most newborns were firstborns (82.64%) and a large number of participants (77.36%) did not plan on having more children (**Figure 1**).

### Pre-pregnancy, prenatal examinations, and fertility circumstances

We observed that older (as opposed to younger: 27.93% vs 18.91%; *P* < 0.001) or wealthier couples (as opposed to less affluent: 25.81% vs 17.89% to approximately 18.73%; *P* < 0.001) were most likely to have carrier screenings before pregnancy. Moreover, the number of women getting these screenings is significantly higher than men (30.25% vs 18.34%, *P* < 0.001). We have also noticed that where a pregnant woman lives influences her choice in pregnancy tests. People in provincial capitals tended to prefer NIPT (as opposed to people in non-capital areas: 58.12% vs 52.91%; *P* < 0.001), while people in non-capital areas showed a preference for Down syndrome screening (as opposed to people in provincial capitals: 25.34% vs 20.54%; *P* < 0.001). Age also emerged as a determinant factor, with women <35 predominantly choosing Down syndrome screening (as opposed to women ≥35 years: 24.79% vs 7.81%; *P* < 0.001) and their older counterparts (≥35 years old) leaning towards NIPT (as opposed to women <35 years old: 67.27% vs 53.62%; *P* < 0.001). Individuals with a high school diploma or lower degree were the least likely to undergo Down syndrome screening or NIPT (10.07% vs 2.98% to approximately 3.27%), while affluent individuals (with earnings >400 000 CNY) showed a preference towards NIPT (61.94% vs 53.25% to approximately 53.33%; *P* < 0.001) (**Table 1**).

**Table 1:** Pre-pregnancy examinations and reproductive history*

	Carrier screening			Pregnancy check				
**Characteristics**	**Yes, n = 482 (20.17)**	**No, n = 1908 (79.83)**	***P*-value**	**Down syndrome screening, n = 536 (22.43)**	**NIPT, n = 1327 (55.52)**	**Both of them, n = 441 (18.45)**	**Unchecked, n = 86 (3.60)**	***P*-value**
Age			<0.001					<0.001
*<35 y*	389 (18.91)	1668 (81.09)		510 (24.79)	1103 (53.62)	385 (18.72)	59 (2.87)	
*≥35 y*	93 (27.93)	240 (72.07)		26 (7.81)	224 (67.27)	56 (16.82)	27 (8.11)	
Gender			<0.001					<0.001
*Male*	371 (18.34)	1652 (81.66)		430 (21.26)	1194 (59.02)	334 (16.51)	65 (3.21)	
*Female*	111 (30.25)	256 (69.75)		106 (28.88)	133 (6.57)	107 (5.29)	21 (1.04)	
Household registration			0.117					<0.001
*Municipality/provincial capital*	361 (21.00)	1358 (79.00)		353 (20.54)	999 (58.12)	304 (17.68)	63 (3.67)	
*Non-municipality/provincial capital*	121 (18.03)	550 (81.97)		183 (25.34)	382 (52.91)	134 (18.56)	23 (3.19)	
Education			0.064					<0.001
*High school degree or below*	32 (23.02)	107 (76.98)		33 (23.74)	68 (48.92)	24 (17.27)	14 (10.07)	
*College degree*	318 (18.92)	1363 (81.08)		382 (22.72)	949 (56.45)	295 (17.55)	55 (3.27)	
*Master degree or above*	132 (23.16)	438 (76.84)		121 (21.23)	310 (54.39)	122 (21.40)	17 (2.98)	
Family income in CNY			<0.001					<0.001
*≤200 000*	204 (17.89)	936 (82.11)		284 (24.91)	607 (53.25)	204 (17.89)	45 (3.95)	
*210 000–300 000*	118 (18.73)	512 (81.27)		151 (23.97)	336 (53.33)	126 (20.00)	17 (2.70)	
*≥310 000*	160 (25.81)	460 (74.19)		101 (16.29)	384 (61.94)	111 (17.90)	24 (3.87)	

NIPT – non-invasive prenatal test

*Presented as n (%) unless specified otherwise.

Most babies in our sample were firstborns (82.64%), and most couples had no plans for more children (77.36%). Older couples (age ≥35) had a higher proportion of second-born children (46.55% vs 11.42%; *P* < 0.001) and desired fewer additional children than younger couples (12.61% vs 24.26%; *P* < 0.001). Moreover, families residing in provincial capitals, those with less educated backgrounds, and those with higher income levels tended to have more children (*P* < 0.001). Notably, females exhibited a stronger desire for additional offspring than males (29.16% vs 21.45%; *P* < 0.001) (Table S3 in the **Online Supplementary Document**).

### Understanding of NBGS among the respondents

Most couples believed they had some understanding of NBGS (68.38%), with an average score of  2.96 (SD = 0.96) on NBGS-related questions (range: 0–4) (**Table 2**). This perception was pronounced among women (*P* < 0.001), individuals with advanced education, high-income earners (*P* < 0.001), those who have undergone carrier screening (*P* < 0.001), and those without Down syndrome or non-invasive screening (*P* < 0.01). However, through the testing of NBGS-related questions, we found that only the highly educated population (master’s degree or above, as opposed to college degree or below: 3.24, SD = 0.86 vs 2.14, SD = 1.04 to approximately 2.93, SD = 0.94; *P* < 0.001) and the high-income population (income of ≥210 000 CNY as opposed to income ≤200 000 CNY: 3.07, SD = 0.92 to approximately 3.14, SD = 0.92 vs 2.80, SD = 0.98; *P* < 0.001) showed consistency between their self-perception and actual scores regarding NBGS. Although the proportion of people who perceived themselves as having a good understanding of NBGS was significantly higher among those who had not undergone Down syndrome screening or non-invasive procedures (*P* < 0.01), their scores were the lowest when answering NBGS-related questions. We also found that, while there was no difference in the self-perception of NBGS among people from various regions, those living in provincial capital cities had a more accurate understanding of NBGS when compared to non-capital city residents (2.99, SD = 0.96 vs 2.88, SD = 0.96; *P* < 0.01).

**Table 2:** Understanding of NBGS among the respondents*

	Q12†				Q13–16†		
**Characteristics**	**Much, n = 669 (28.00)**	**Little, n = 965 (40.38)**	**No, n = 756 (31.63)**	***P*-value**	**Score (SD)‡**	***P*-value**	***P*-value – Dunn’s test**
Age				0.670		0.939	
*<35 y*	569 (27.66)	834 (40.54)	654 (31.79)		2.96 (0.94)		
*≥35 y*	100 (30.03)	131 (39.34)	102 (30.63)		2.94 (1.05)		
Gender				<0.001		0.463	
*Male*	520 (25.70)	839 (41.47)	664 (32.82)		2.91 (1.02)		
*Female*	149 (40.60)	126 (34.33)	92 (25.07)		2.96 (0.95)		
Household registration				0.475		0.006	
*Municipality/provincial capital*	505 (29.38)	676 (39.33)	538 (31.30)		2.99 (0.96)		
*Non-municipality/provincial capital*	164 (24.44)	289 (43.07)	218 (32.49)		2.88 (0.96)		
Education				0.002		<0.001	
*High school degree or below*	33 (23.74)	61 (43.88)	45 (32.37)		2.14 (1.04)		AB<0.001
*College degree*	452 (26.89)	662 (39.38)	567 (33.73)		2.93 (0.94)		AC<0.001
*Master's degree or above*	184 (32.28)	242 (42.46)	144 (25.26)		3.24 (0.86)		BC<0.001
Family income in CNY				<0.001		<0.001	
*≤200 000*	295 (25.88)	465 (40.79)	380 (33.33)		2.80 (0.98)		AB<0.001
*210 000–300 000*	160 (25.40)	259 (41.11)	211 (33.49)		3.07 (0.92)		AC<0.001
*≥310 000*	214 (34.51)	241 (38.87)	165 (26.61)		3.14 (0.92)		
Carrier screening				<0.001		0.183	
*Yes*	212 (43.98)	166 (34.44)	104 (21.58)		3.01 (0.94)		
*No*	457 (23.95)	799 (41.88)	652 (34.17)		2.95 (0.96)		
Pregnancy check				0.008		0.910	
*Down syndrome screening*	140 (26.12)	197 (36.75)	199 (37.13)		2.96 (1.02)		
*Non-invasive prenatal testing*	374 (28.18)	556 (41.90)	397 (29.92)		2.97 (0.94)		
*Both of them*	122 (29.68)	188 (45.74)	131 (31.87)		2.96 (0.95)		
*Unchecked*	33 (38.37)	24 (27.91)	29 (33.72)		2.91 (0.93)		
Which baby is this				0.027		0.104	
*First*	537 (27.19)	806 (40.81)	632 (32.00)		2.97 (0.95)		
*Second*	127 (32.56)	143 (36.67)	120 (30.77)		2.94 (0.97)		
*Third or more*	5 (20)	16 (64)	4 (16.00)		2.4 (1.41)		
Plan to have more baby				0.077		0.174	
*Yes*	172 (31.79)	204 (37.71)	165 (30.50)		2.92 (0.93)		
*No*	497 (26.88)	761 (41.16)	591 (31.96)		2.97 (0.97)		

SD – standard deviation

*Presented as n (%) unless specified otherwise.

†Q12 – ‘Are you and your spouse familiar with NBGS?,’ Q13 – ‘Is it true that couples with no family history of genetic diseases will not have children with genetic diseases?,’ Q14 – ‘Does a genetic screening result of ‘not detected’ mean that the child will not have any possibility of having genetic or other congenital diseases?,’ Q15 – ‘Do you know what ‘carrier’ means in the context of genetic screening results?,’ Q16 – ‘Do you know what it means if NBGS shows the possibility of having a disease?.’

‡Score – total sum of scores for Q13 to Q16, presented as x̄ (SD) (range: 0–4).

### Choices made by respondents based on the results of NBGS

For newborns identified as carriers of pathogenic genes through NBGS, 98.95% of the parents still wanted to know the results, even if they were unrelated to the phenotype or may have caused anxiety within the family (**Table 3**). Among them, the proportion of women who did not wish to know the results was slightly higher than that of men (1.91% vs 0.89%; *P* < 0.05). For screening results unrelated to phenotype, the highly educated population were more likely to choose not to be informed than the less educated ones (*P* < 0.05). When being informed of carrier results may lead to family anxiety, a higher proportion of couples who had not undergone Down syndrome screening or NIPT preferred not to be informed compared to those who had (10.47% vs 2.64% to approximately 3.85%; *P* < 0.01). Moreover, a higher proportion of people planning to have more children did not wish to know the carrier results compared to those without plans for further reproduction (5.55% vs 2.70%; *P* < 0.01). Encouragingly, after learning that the baby is a ‘carrier,’ 98.83% of couples declared willingness to have prenatal counselling before reproducing again, while 91.63% stated that they would inform their child about carrier status after it reached adulthood, especially among the high-income population (*P* < 0.05) and those who have undergone prenatal carrier screening (*P* < 0.01).

**Table 3:** Choices made by respondents based on the results of NBGS*

	Q17†				Q18†			Q19†			Q20†			Q21†		
**Characteristics**	**YesF, n = 1065, (44.5)**	**YesNF, n = 1300 (54.39)**	**No, n = 25 (1.05)**	***P*-value**	**Yes n = 2283, (95.5)**	**No, n = 107 (9.73)**	***P*-value**	**Yes, n = 2310 (96.6)**	**No, n = 80 (3.35)**	***P*-value**	**Yes, n = 2362 (98.8)**	**No, n = 8 (0.33)**	***P*-value**	**Yes, n = 2190 (91.6)**	**No, n = 200 (8.37)**	***P*-value**
Age				0.933			0.459			0.589			0.527			0.473
*<35 y*	915 (44.48)	1121 (54.50)	21 (1.02)		1968 (95.67)	89 (4.32)		1986 (96.54)	71 (3.45)		2030 (98.69)	8 (0.39)		1881 (91.44)	176 (8.56)	
*≥35 y*	150 (45.05)	179 (53.75)	4 (1.20)		315 (94.59)	18 (5.41)		324 (97.30)	9 (2.70)		332 (99.70)	0 (0.00)		309 (92.79)	24 (7.21)	
Gender				0.0384			0.019			0.050			0.474			1
*Male*	919 (45.43)	1086 (53.68)	18 (0.89)		1941 (95.95)	82 (4.05)		1962 (96.98)	61 (3.02)		1998 (98.76)	8 (0.40)		185 (91.65)	169 (8.35)	
*Female*	146 (39.78)	214 (58.31)	7 (1.91)		342 (93.19)	25 (6.81)		348 (94.82)	19 (5.18)		364 (99.18)	0 (0.00)		336 (91.55)	31 (8.45)	
Household registration				0.752			0.588			0.442			0.838			0.062
*Municipality/provincial capital*	759 (44.15)	941 (54.74)	19 (1.11)		1645 (95.70)	74 (4.30)		1665 (96.86)	54 (3.14)		1701 (98.95)	5 (0.29)		1587 (92.32)	132 (7.68)	
*Non-municipality/provincial capital*	306 (45.60)	359 (53.50)	6 (0.89)		638 (95.08)	33 (4.92)		645 (96.13)	26 (3.87)		661 (98.51)	3 (0.45)		603 (89.87)	68 (10.13)	
Education				0.054			0.024			0.202			0.512			0.937
*High school degree or below*	59 (42.44)	80 (57.55)	0 (0.00)		135 (97.12)	4 (2.88)		131 (94.24)	8 (5.76)		136 (97.84)	1 (0.72)		127 (91.37)	12 (8.6 3)	
*College degree*	774 (46.04)	892 (53.06)	15 (8.92)		1615 (96.07)	66 (3.93)		1630 (96.83)	51 (3.03)		1664 (98.99)	5 (0.30)		1546 (91.97)	135 (8.03)	
*Master degree or above*	232 (40.70)	328 (57.54)	10 (1.75)		533 (93.51)	37 (6.49)		549 (96.32)	21 (3.68)		562 (98.60)	2 (0.35)		522 (91.58)	48 (8.42)	
Family income in CNY				0.568			0.596			0.915			0.218			0.029
*≤200 000*	503 (44.12)	629 (55.18)	8 (0.70)		1094 (95.96)	46 (4.04)		1100 (96.49)	40 (3.51)		1123 (98.51)	5 (0.44)		1040 (91.23)	100 (8.77)	
*210 000–300 000*	285 (45.24)	337 (53.49)	8 (1.27)		600 (95.24)	30 (4.76)		610 (96.83)	20 (3.17)		627 (99.52)	0 (0.00)		567 (90.00)	63 (10.00)	
*≥310 000*	277 (44.68)	334 (53.8)	9 (1.45)		589 (95.00)	31 (5.00)		600 (96.77)	20 (3.23)		612 (98.71)	3 (0.48)		583 (94.03)	37 (5.97)	
Carrier screening				0.313			1			1			0.325			0.006
*Yes*	200 (41.49)	277(57.47)	5 (1.04)		460 (95.44)	22 (4.56)		466 (96.68)	16 (3.32)		479 (99.38)	0 (0.00)		457 (94.81)	25 (5.19)	
*No*	865 (45.34)	1023 (53.62)	20 (1.05)		1823 (95.55)	85 (4.45)		1844 (96.65)	64 (3.35)		1883 (98.69)	8 (0.42)		1733 (90.83)	175 (9.17)	
Pregnancy check				0.138			0.912			0.001			0.849			0.891
*Down syndrome screening*	260 (48.51)	272 (50.75)	4 (0.75)		514 (95.90)	22 (4.10)		517 (96.46)	19 (3.54)		528 (98.51)	2 (0.37)		490 (91.42)	46 (8.58)	
*NIPT*	584 (44.01)	730 (55.01)	13 (0.98)		1268 (95.55)	59 (4.45)		1292 (97.36)	35 (2.64)		1315 (99.1)	4 (0.30)		1219 (91.86)	108 (8.14)	
*Both of them*	190 (43.08)	243 (55.10)	8 (1.81)		419 (95.01)	22 (4.99)		424 (96.15)	17 (3.85)		434 (98.41)	2 (0.45)		401 (90.93)	40 (9.07)	
*Unchecked*	31 (36.05)	55 (63.95)	0 (0.00)		82 (95.35)	4 (4.65)		77 (89.53)	9 (10.47)		85 (98.84)	0 (0.00)		80 (93.02)	6 (6.98)	
Which baby is this				0.015			0.179			0.334			0.030			0.009
*First*	884 (44.76)	1073 (54.33)	18 (0.91)		1887 (95.54)	88 (4.46)		1910 (96.71)	65 (3.29)		1954 (98.94)	5 (0.25)		1801 (91.19)	174 (8.81)	
*Second*	171 (43.85)	215 (55.13)	4 (1.03)		374 (95.90%)	16 (4.10)		377 (96.67)	13 (3.33)		386 (98.97)	2 (0.51)		369 (94.62)	21 (5.38)	
*More*	10 (40.00)	12 (48.00)	3 (12.00)		2 (88.00)	3 (12.00)		23 (92.00)	2 (8.00)		22 (88.00)	1 (4.00)		20 (80.00)	5 (20.00)	
Plan to have more baby				0.162			0.865			0.002			0.794			0.456
*Yes*	250 (46.21)	282 (52.13)	9 (1.66)		518 (95.75)	23 (4.25)		511 (94.45)	30 (5.55)		535 (98.89)	1 (0.18)		491 (90.76)	50 (9.24)	
*No*	815 (44.08)	1018 (55.06)	16 (0.87)		1765 (95.46)	84 (4.54)		1799 (97.30)	50 (2.70)		1827 (98.81)	7 (0.38)		1699 (91.89)	150 (8.11)	

NIPT – non-invasive prenatal test, YesF – yes and face to face, YesNF – yes and non-face to face

*Presented as n (%) unless specified otherwise.

†Q17 – ‘If your child’s NBGS result is ‘carrier’, would you like to be informed?,’ Q18 – ‘If your child’s NBGS result shows that they are a carrier of a pathogenic gene but it is most likely unrelated to clinical symptoms, meaning there is a low probability of them developing the disease, would you still want the hospital to inform you in the report?,’ Q19 – ‘Would you still want to know about the ‘carrier’ result if knowing your child’s NBGS result may lead to anxiety in your family’s future life?,’ Q20 – ‘If your child’s NBGS results show they are a ‘carrier’, would you consider having prenatal counselling before having another child?,’ Q21 – ‘If you find out that your child is a ‘carrier’ of a pathogenic gene through NBGS, would you inform them after they become an adult?.’

### Impact of being a carrier on the family

Most couples (98.74%) considered NBGS necessary for early detection and treatment of potential diseases. However, in families with multiple children, the proportion of individuals considering NBGS less essential was higher (16.00% vs 0.91 to approximately 2.05%) (**Table 4**). Most families believed NBGS has had a positive impact on their future life (73.18%) by providing clearer insights into their child’s health, which makes their family life more stable and reassuring. Conversely, a small fraction (19.16%) believed NBGS could cause stress and anxiety, particularly prevalent amongst those less educated (high school degree or below as opposed to a college degree or above: 23.02% vs 16.67% to approximately 19.69%; *P* < 0.01).

**Table 4:** Impact of being a carrier on the family*

	Think it is necessary to perform NBGS			The impact of NBGS results to your family			
**Characteristics**	**Yes, n = 2360 (98.74)**	**No, n = 30 (1.26)**	***P*-value**	**Positive, n = 1749 (73.18)**	**Negative, n = 458 (19.16)**	**None, n = 143 (5.98)**	***P*-value**
Age			0.865				0.577
*<35 y*	2032 (98.78)	25 (1.22)		1505 (73.16)	397 (19.30)	119 (5.79)	
*≥35 y*	328 (98.50)	5 (1.50)		244 (73.27)	61 (18.32)	24 (7.21)	
Gender			1				<0.001
*Male*	1998 (98.76)	25 (1.24)		1496 (73.95)	396 (19.57)	98 (4.84)	
*Female*	362 (98.64)	5 (1.36)		253 (68.94)	62 (16.89)	45 (12.26)	
Household registration			0.396				0.506
*Municipality/provincial capital*	1700 (98.89)	19 (1.11)		1257 (73.12)	327 (19.02)	109 (6.34)	
*Non-municipality/provincial capital*	660 (98.36)	11 (1.64)		492 (73.32)	131 (19.52)	34 (5.07)	
Education			0.650				<0.001
*High school degree or below*	138 (99.28)	1 (0.72)		88 (63.31)	32 (23.02)	14 (10.07)	
*College degree*	1661 (98.81)	20 (1.19)		1244 (74.00)	331 (19.69)	79 (4.70)	
*Master degree or above*	561 (98.42)	9 (1.58)		417 (73.16)	95 (16.67)	50 (8.77)	
Family income in CNY			0.346				0.734
*≤200 000*	1129 (99.04)	11 (0.96)		828 (72.63)	227 (19.91)	62 (5.44)	
*210 000-300 000*	622 (98.73)	8 (1.27)		469 (74.44)	114 (18.10)	40 (6.35)	
*≥310 000*	609 (98.23)	88 (14.19)		452 (72.90)	117 (18.87)	41 (6.61)	
Carrier screening			0.801				0.064
*Yes*	477 (98.96)	5 (1.04)		361 (74.90)	79 (16.39)	37 (7.68)	
*No*	1883 (98.69)	25 (1.31)		1388 (72.75)	379 (19.86)	106 (5.56)	
Pregnancy check			0.741				<0.001
*Down screening*	527 (98.32)	9 (1.68)		393 (73.32)	102 (19.03)	32 (5.97)	
*Non-invasive prenatal testing*	1313 (98.94)	14 (1.06)		965 (72.72)	273 (20.57)	66 (4.97)	
*Both of them*	435 (98.64)	6 (1.36)		332 (75.28)	71 (16.10)	31 (7.03)	
*Unchecked*	85 (98.84)	1 (1.16)		59 (68.60)	12 (13.95)	14 (16.28)	
Which baby is this			<0.001				0.111
*First*	1957 (99.09)	18 (0.91)		1455 (73.67)	368 (18.63)	118 (5.97)	
*Second*	382 (97.95)	8 (2.05)		282 (72.31)	81 (20.77)	23 (5.90)	
*More*	21 (84.00)	4 (16.00)		12 (48.00)	9 (36.00)	2 (8.00)	
Plan to have more baby			0.898				0.080
*Yes*	535 (98.89)	6 (1.11)		400 (73.94)	88 (16.27)	43 (7.95)	
*No*	1825 (98.70)	24 (1.30)		1349 (72.96)	370 (20.01)	100 (5.41)	

NBGS – newborn genetic screening

*Presented as n (%) unless specified otherwise.

We further explored the benefits and challenges that NBGS brought to their life through open-ended questions and found that people generally felt that NBGS improved their understanding of their baby’s health and knowledge of genetic diseases. Families with ill children thought NBGS could enhance the child’s life quality through early detection and treatment. However, they also expressed concerns, including potential genetic information exposure leading to social issues like discrimination and family distress due to results possibly affecting familial bonds. Furthermore, they also provided constructive suggestions for current NBGS, such as incorporating NBGS into medical insurance, which can assist in future improvements and optimisation (Table S4 in the **Online Supplementary Document**).

## DISCUSSION

Our study provides the first in-depth understanding of the views of parents whose newborns underwent NBGS and were reported as carriers, including their opinions on NBGS and the disclosure of carrier status; on whether NBGS carrier information should be reported; and the impact on their emotions, family life, and social relationships after receiving NBGS carrier results. We found that participants generally prefer learning about their carrier status and tend to maintain an optimistic attitude, which highlights the feasibility of reporting carrier results. We also found that individuals who have undergone prenatal carrier screening exhibit a greater understanding of NBGS compared to other groups. However, those who have not done so, actually harbour many misconceptions and show little interest in NBGS results, despite perceiving themselves as familiar with NBGS (**Table 2**, **Table 3**). This suggests that prenatal screening can increase parents’ understanding of genetic diseases and that it is meaningful in promoting the implementation of NBGS, which means that education on prenatal screening is equally important as education on NBGS, especially in carrier screening. By promoting awareness through education, we can help the public better understand and actively participate in NBGS.

We observed that men and women hold different attitudes toward the results of NBGS (Table S7 in the **Online Supplementary Document**): Women tend to be more emotional, showing more pronounced psychological fluctuations and a tendency towards anxiety, while men tend to be more rational. This suggests that, when it comes to communicating results in the subsequent clinical implementation of NBGS, priority may be given to informing the child’s father. Although both NBGS and carrier screening are screening projects, they have distinct differences [28–30] (Table S5 in the **Online Supplementary Document**). However, they are not independent, but interconnected. Currently, the percentage of individuals who have undergone carrier screening is relatively low (20.17%). Our findings not only suggest a positive impact of carrier screening on advancing NBGS implementation (Table S6 in the **Online Supplementary Document**), but also highlight NBGS’s potential to promote the advancement of carrier screening. In cases where clinical findings indicate that parents are carriers of diseases with a relatively high incidence based on NBGS results, and in cases where they plan to have more children, it is recommended that clinical genetic counsellors encourage parents to enhance carrier screening. This helps to determine whether both parents of the newborn are carriers of high-incidence diseases, thereby reducing the risk of the child developing illnesses in future pregnancies.

We further found significant differences in the choices of prenatal screening and the awareness of NBGS between the provincial capitals/direct-controlled municipalities and non-provincial capital populations, which indicated that cultural and socio-economic backgrounds play an equally pivotal role in NBGS implementation. Provincial capitals/direct-controlled municipalities typically have denser populations, more developed infrastructure, faster-paced lifestyles, higher incomes, and higher consumption levels compared to non-provincial capitals. In our study, they also had a higher prevalence of NIPT adoption, while Down syndrome screening was more frequent in non-provincial capitals (**Table 1**). We speculate that this might be due to the wider popularity of NIPT in provincial capitals/direct-controlled municipalities and the combined consideration of NIPT costs and population income levels. People in provincial capitals, who also have higher income levels, are more likely to afford the higher testing costs of NIPT. Additionally, the population in provincial capitals exhibits a higher level of accuracy in understanding NBGS compared to non-provincial capitals (**Table 2**). This could be attributed to the relatively advanced socioeconomic status and concentrated talent pool in provincial capitals, contributing to a generally heightened level of comprehensive awareness in the population. Our findings suggest that countries and cities at different development levels may differ in the awareness and choices regarding NBGS due to varying cultural and socio-economic backgrounds. These insights offer guidance for the implementation of NBGS in countries and cities with differing development levels. For example, prioritising NBGS may be more feasible in countries with higher development levels, i.e. in developed or even developing countries with first-tier or second-tier cities. In contrast, in developing countries or slower-developing third-tier or fourth-tier cities, emphasis may be placed on enhancing public education to raise overall awareness before gradually introducing and popularising NBGS, making it more widely accepted by the public.

The debate over whether to disclose carrier results from NBGS has been ongoing. The American Society of Human Genetics explicitly recommends keeping non-critical gene test results confidential or deferring the decision to disclose until the child reaches adulthood [31]. Meanwhile, genetic counsellors advocate for disclosing carrier information, emphasising its importance for family reproductive choices and family planning, even if the infant may not show signs of the disease [20]. Our research indicates that, from the parents' perspective, most tend to prefer knowing their child’s carrier status, even if it causes anxiety within the family. Additionally, our clinical observations show that when parents receive NBGS carrier results, many choose to seek genetic counselling through phone consultations or hospital visits [32]. This underscores the significance parents attribute to NBGS carrier results while emphasizing the vital role of clinical counselling. Misinterpreting NBGS carrier results can easily lead to negative issues such as family anxiety. Therefore, clinical practitioners must continually enhance their communication skills in genetic counselling, using clear and effective communication to mitigate the risks of anxiety, stress, shame, and misunderstandings associated with discovering carrier status [33]. Simultaneously, we recognise that the disclosure of carrier pathogenic information may lead to privacy breaches and family concerns [21,22]. Hence, it is essential to respect the opinions of family members to the greatest extent possible. Those who prefer not to know carrier information can consider choosing not to be informed when signing the informed consent, thereby avoiding the potential negative impact on these family members upon learning about carrier screening results.

Due to the current exclusive implementation of the clinical NBGS project in Nanjing, Jiangsu Province, the research scope of our study was restricted to the population who underwent NBGS in Nanjing, Jiangsu Province. Therefore, our findings can largely represent the views of the population within the Jiangsu region and may not encompass the opinions of populations in different provinces and cities. Additionally, the limited number of collected questionnaires may introduce some degree of bias to the research findings. In the future, it is necessary to conduct a large-scale survey across multiple provinces and cities after the implementation of NBGS in more regions to validate and enhance the results of this study.

## CONCLUSIONS

Our research shows that in NBGS, most parents are interested in carrier results, even when unrelated to clinical symptoms or causing family anxiety. Specifically, those with a thorough understanding of NBGS – such as individuals with higher education and income, residents of provincial capitals, and those undergoing carrier screening before pregnancy – tend to hold a positive attitude towards NBGS results. They tend to seek prenatal counselling before subsequent pregnancies and share carrier status with their children upon reaching adulthood. Conversely, individuals with lower education levels are the primary group exhibiting a negative attitude. Our survey is the first to explore the perspectives of NBGS carrier families in China, highlighting population differences in attitudes towards disclosing NBGS carrier information. This knowledge is valuable for targeted improvements in NBGS in China and serves as a reference for its implementation globally.

## Additional material


Online Supplementary Document
