# Supplementary material for: Utility, benefits, and risks of newborn genetic screening carrier reports for families
Source: J Glob Health. 2024 Feb 23;14:04044. doi: 10.7189/jogh.14.04044 (PMC10884785; doi:10.7189/jogh.14.04044)
Supplement: Online Supplementary Document [file jogh-14-04044-s001.pdf]

Online Supplementary Document

Table S1. Questionnaire designed.

No.	Question
Q1	Your household registration
Q2	Your Age
Q3	Your gender.
Q4	Your education levels.
Q5	Your family income:
Q6	Did you and your partner undergo carrier screening before or during pregnancy?
Q7	Did you or your spouse have a Down’s screening or non-invasive prenatal testing during pregnancy?
Q8	Did you or your spouse have an amniocentesis during pregnancy?
Q9	Did you or your spouse have any fetal abnormalities during pregnancy?
Q10	Which baby is this for you? (e.g., first, second, etc.)
Q11	Do you and your spouse plan to have more children?
Q12	Are you and your spouse familiar with NBGS?
Q13	Is it true that couples with no family history of genetic diseases will not have children with genetic diseases?
Q14	Does a genetic screening result of “not detected” mean that the child will not have any possibility of having genetic or other congenital diseases?
Q15	Do you know what “carrier” means in the context of genetic screening results?
Q16	Do you know what it means if NBGS shows the possibility of having a disease?
Q17	If your child’s NBGS result is “carrier”, would you like to be informed?
Q18	If your child’s NBGS result shows that they are a carrier of a pathogenic gene but it is most likely unrelated to clinical symptoms, meaning there is a low probability of them developing the disease, would you still want the hospital to inform you in the report?
Q19	Would you still want to know about the “carrier” result if knowing your child’s NBGS result may lead to anxiety in your family’s future life?
Q20	If your child’s NBGS results show they are a “carrier”, would you consider having prenatal counseling before having another child?
Q21	If you find out that your child is a “carrier” of a pathogenic gene through NBGS, would you inform them after they become an adult?

Q22	Do you think it's necessary to perform NBGS to identify the genetic causes of diseases in affected children and to intervene and treat them as early as possible?
Q23	What impact do your child's NBGS results have on you and your family's future life?
Q24	What benefits could NBGS offer to you and your family?
Q25	Do you have any concerns regarding the implementation of NBGS?
Q26	Do you have any suggestions or opinions about NBGS?

a, NBGS, newborn genetic screening.

Table S2. Demographic characteristics of respondent.

Characteristics, n (%)	N = 2390
Age	
< 35 years	2057 (86.07%)
≥ 35 years	333 (13.93%)
Gender	
Male	2023 (84.64%)
Female	367 (15.36%)
Household registration	
Municipality / provincial capital	1719 (71.92%)
Non-municipality / provincial capital	671 (28.08%)
Education	
High school degree or below	139 (5.82%)
College degree	1681 (70.33%)
Master degree or above	570 (23.85%)
Family income	
≤200 thousands	1140 (47.70%)
210-300 thousands	630 (26.40%)
≥310 thousands	620 (25.90%)

Table S3. Family condition and planning.

Characteristics, n (%)	Which baby is this			*P*-value	Plan to have more baby		*P*-value
	1 (1975, 82.64%)	2 (390, 16.32%)	≥3 (25, 1.05%)		Yes (541, 22.64%)	No (1849, 77.36%)	
Age				<0.001			<0.001
< 35 years	1808 (87.89%)	235 (11.42%)	14 (0.68%)		499 (24.26%)	1558 (75.74%)	
≥ 35 years	167 (50.15%)	155 (46.55%)	11 (3.30%)		42 (12.61%)	291 (87.39%)	
Gender				0.805			0.001
Male	1668 (82.45%)	333 (16.46%)	22 (1.09%)		434 (21.45%)	1589 (78.55%)	
Female	307 (83.65%)	57 (15.53%)	3 (0.82%)		107 (29.16%)	260 (70.84%)	
Household registration				<0.001			0.043
Municipality / provincial capital	1385 (80.57%)	320 (18.62%)	14 (0.81%)		370 (21.52%)	1349 (78.48%)	
Non-municipality / provincial capital	590 (87.93%)	70 (10.43%)	11 (1.64%)		171 (25.48%)	500 (74.52%)	
Education				<0.001			0.086
High school degree or below	88 (63.31%)	43 (30.94%)	8 (5.76%)		32 (23.02%)	107 (76.98%)	
College degree	1408 (83.76%)	261 (15.53%)	12 (0.71%)		361 (21.48%)	1320 (78.52%)	
Master degree or above	479 (84.03%)	86 (15.09%)	5 (0.88%)		148 (25.96%)	422 (74.04%)	
Family income				<0.001			0.053
≤200 thousands	949 (83.25%)	180 (15.79%)	11 (0.96%)		246 (21.58%)	894 (78.42%)	
210-300 thousands	547 (86.83%)	78 (12.38%)	5 (0.79%)		133 (21.11%)	497 (78.89%)	
≥310 thousands	479 (77.26%)	132 (21.29%)	9 (1.45%)		162 (26.13%)	458 (73.87%)	

Table S4. Benefits, concerns and suggestions in participants' open-end responses.

Theme
**Benefits**
Making family members more informed about their child's health status.
Enhancing the understanding of genetic diseases among family members.

Increased variety and number of genetic diseases that can be detected.
Capable of detecting and treating diseases at an early stage, improving the child's quality of life.

Concerns

Unaffordable high costs.
The exposure of genetic information privacy.
the child might face discrimination due to genetic information.
Knowing the results might cause negative emotions within the family.

Suggestions

Included in medical insurance
Pay attention to privacy protection
Lowering the costs
Nationwide popularization
Increase publicity and explanation
Broader testing diseases
More precise test results

Table S5. Differences between carrier screening and NBGS.

Difference	Carrier screening	NBGS
Objective	Couple	Newborn
Sample source	Peripheral Blood	Dried blood spot
Number of diseases	≤20	>150
Pathogenetic variants	≤448	>10,000
Detection method	Capillary electrophoresis-based multiplex PCR assay/ NGS	Targeted capture-based NGS
Detection duration	4 weeks	2 weeks
Intervention measures	Genetic counseling	Treatment or follow-up
Cost	≈ 800~1600 RMB/couple	≈ 850 RMB/newborn

Table S6. Effect of Carrier Screening on NBGS by Education Levels.

Characteristics, n (%)	Q12				Q13-16		Q21			Q23			
	Much	Little	No	*P*-value	Score	*P*-value	Yes	No	*P*-value	Positive	Negative	None	*P*-value
High school degree or below				0.0302		0.8967			0.1377				0.2815
Carrier screening done	12 (37.5%)	15 (46.88%)	5 (15.63%)		2.16 (±1.14)		31 (96.88%)	1 (3.13%)		22 (68.75%)	9 (28.13%)	1 (3.13%)	
No carrier screening done	21 (19.63%)	46 (42.99%)	40 (37.38%)		2.13 (±1.01)		91 (85.05%)	16 (14.95%)		66 (64.71%)	23 (22.55%)	13 (12.75%)	
College degree				<0.001		0.0447			0.01142				0.3049
Carrier screening done	130 (40.88%)	109 (34.28%)	79 (24.84%)		3.03 (±0.92)		304 (95.60%)	14 (4.40%)		234 (74.76%)	59 (18.85%)	20 (6.39%)	
No carrier screening done	322 (23.62%)	553 (40.57%)	488 (35.80%)		2.91 (±0.95)		1242 (91.12%)	121 (8.88%)		1010 (75.32%)	272 (20.28%)	59 (4.40%)	
Master degree or above				<0.001		0.2563			0.8257				0.006166
Carrier screening done	70 (53.03%)	42 (31.82%)	20 (15.15%)		3.18 (±0.85)		122 (92.42%)	10 (7.58%)		105 (79.55%)	11 (8.33%)	16 (12.12%)	
No carrier screening done	114 (26.03%)	200 (45.66%)	124 (28.31%)		3.26 (±0.86)		400 (91.32%)	38 (8.68%)		312 (72.56%)	84 (19.53%)	34 (7.91%)	

Table S7. Different considerations between males and females

Characteristics, n (%)	Male N=2023 (84.64%)	Female N=367 (15.36%)	*P*-value
Are you and your spouse familiar with NBGS?			<0.001
Much	520 (25.70%)	149 (40.60%)	

Little	839 (41.47%)	126 (34.33%)	
No	664 (32.82%)	92 (25.07%)	
If your child's NBGS result is "carrier", would you like to be informed?			0.038
Yes, face to face	919 (45.43%)	146 (39.78%)	
Yes, non-face to face	1086 (53.68%)	214 (58.31%)	
No	18 (0.89%)	7 (1.91%)	
Would like to be informed if the child is a "carrier" even if there is a low probability of developing the disease?			0.019
Yes	1941 (95.95%)	342 (93.19%)	
No	82 (4.05%)	25 (6.81%)	
Still want to know the "carrier" result even if it may lead to anxiety in your family's future life?			0.050
Yes	1962 (96.98%)	348 (94.82%)	
No	61 (3.02%)	19 (5.18%)	
Consider having prenatal counseling before having another child if this child is a "carrier"?			0.474
Yes	1998 (98.76%)	364 (100%)	
No	8 (0.40%)	0 (0.00%)	
Q21			1
Yes	1854 (91.65%)	336 (91.56%)	
No	169 (8.35%)	31 (8.45%)	
Think it is necessary to perform NBGS			1
Yes	1998 (98.76%)	362 (98.64%)	
No	25 (1.24%)	5 (1.36%)	
The impact of NBGS results to your family			<0.001
Positive	1496 (73.95%)	253 (68.94%)	
Negative	396 (19.57%)	62 (16.89%)	
None	98 (4.84%)	45 (12.26%)	
